# Methionine supplementation improves reproductive performance, antioxidant status, immunity and maternal antibody transmission in breeder Japanese quail under heat stress conditions

**DOI:** 10.5194/aab-62-275-2019

**Published:** 2019-05-14

**Authors:** Omid Kalvandi, Amirali Sadeghi, Ahmad Karimi

**Affiliations:** Department of Animal Science, Faculty of Agriculture, University of Kurdistan, Sanandaj, Iran

## Abstract

This study was conducted to determine the effects of methionine (Met) supplementation on productive and reproductive performance, immune response and antioxidant status in breeder quails reared under heat stress (HS). A total of 125 breeder quails were divided into five groups. One group was kept in an environmentally controlled room at 22 ${}^{\circ }$C and considered as thermoneutral, and four groups were kept at 34 ${}^{\circ }$C and fed a basal diet (heat stressed) or a basal diet with Met concentrations 1.15, 1.30 and 1.45 times the quail requirements per NRC (1994) recommendations. HS decreased egg production in birds fed the basal diet (P<0.05). Higher feed intake (P<0.05), egg production (P<0.05), improved feed efficiency (P<0.05) and Haugh unit and hatchability variables (P<0.05) occurred in Met supplemented groups. Birds receiving Met under HS had higher maternal serum IgG, egg yolk IgY and offspring serum IgG (mg mL${}^{-1}$). Quails receiving the Met supplementation diets exhibited higher (P<0.05) plasma levels and liver activity of superoxide dismutase, catalase and glutathione peroxidase as well as lower (P<0.05) plasma and liver levels of malondialdehyde compared to the HS group fed the basal diet. All breeder quails receiving the Met supplement had lower (P<0.05) heterophil and H/L ratios as well as higher (P<0.05) lymphocytes than quails fed the basal diet under the same stress conditions. Our results suggest that dietary supplementation with Met could improve the performance, immunity and antioxidant status of quails by reducing the negative effects of HS.

## 1 Introduction

Heat as a stressor for birds has been studied extensively for many years. Heat stress (HS) occurs when the amount of heat produced by an animal exceeds the animal's capacity to dissipate the heat to its surrounding environment. Birds experiencing HS tend to reduce their heat production by limiting feed intake, with subsequent negative effects on live weight gain, egg production, egg quality and feed efficiency (Donkoh, 1989), as well as a negative effect on the productive performance of poultry. Several techniques are available to alleviate the adverse effect of HS on poultry performance. Because of the high costs associated with cooling structures that house animals, solutions have been aimed primarily at dietary manipulation (Konca et al., 2009). Nutritional strategies have included the following: adjusting feeding times or temporary feed withdrawal (Francis et al., 1990), dietary energy and protein levels manipulation (Austic, 1985), diet supplementation with vitamin C (Carmina and Cristina, 2013), Se or Cr (Habibian et al., 2014; Ghazi et al., 2012), probiotics and prebiotics (Berrin, 2011), essential oils (Dalkilici et al., 2015) and water supplementation with electrolytes (Lara and Rostagno, 2013).

Recently, the connection between HS and oxidative stress has received much interest (Mujahid et al., 2005, 2009). Oxidative stress is the presence of reactive species in excess of the available antioxidant capacity animal cells. The effects of HS are possibly due to acceleration in the rate of reactive oxygen species (ROS) formation and/or an increase in ROS reactivity (Bai et al., 2003). The defenses of organisms against ROS may be mediated by nonenzymatic or enzymatic antioxidants, mainly represented by the enzymes superoxide dismutase (SOD), catalase (CAT) and the glutathione (GSH) defense system (Abrashev et al., 2008). Antioxidant mechanisms are more often attributed to vitamins (e.g., vitamin E and vitamin C) than to amino acids. However, sulfur amino acids (SAAs) play a major role in the antioxidant systems of the cell in different ways.

Methionine (Met) is a methyl group donor and a precursor of cysteine, cystathionine, homocysteine, S-adenosylhomocysteine and glutathione (Rubin et al., 2007). Diets with Met deficiency result in an increase in heat production (Sekiz et al., 1975), followed by an increase in adverse effects of HS when the environmental temperature is high (Bunchasak and Silapasorn, 2005). Balancing the amino acid composition in the diet with Met supplementation improves production performance through pathways of polyamine metabolism (Gonzalez-Esquerra and Leeson, 2005) and glutathionine (derived from methionine) and may reduce the damage from oxidative stress. Also, the Met requirement determined by NRC (1994) is for laying quails and this may be insufficient for supporting the reproductive performance and hatchability in breeder quails (especially under hyper-thermoneutral conditions). It has been shown that dietary supplementation of Met above NRC (1994) recommendation enhanced cellular immunity in birds (Tsiagbe et al., 1987). Therefore, dietary supplementation of breeder diets with Met above the NRC recommendations may increase the transmission of antibodies to offspring in breeders.

There is very limited information in the literature on the use of Met in breeder quails. Therefore, the aim of the present study was to determine whether dietary supplementation with Met could reduce the adverse effect of HS on the performance, antioxidant status and immune response, as well as the transfer rate of maternal IgY to offspring (no report in the literature) in heat-stressed breeder quails.

## 2 Materials and methods

All experimental protocols adhered to the guidelines of, and were approved by, the Animal Ethics Committee of the University of Kurdistan (approval code 32155/1395; Sanandaj, Iran).

### 2.1 Experimental design and diets

A total of 200 Japanese quails at 45 d of age were obtained from a local supplier. The obvious runts and birds with extreme weight were eliminated. Overall, 125 Japanese quails at 45 d of age were distributed in a completely randomized design, consisting of five treatments, five replicates and five (four females and one male) birds per replicate. A period of 20 d was provided for the birds to adapt to the basal diets, cages and a thermoneutral environmental temperature (22 ${}^{\circ }$C). The relative humidity was maintained between 55 % and 60 %. The birds were exposed to a photoperiod of 16 h of light and 8 h of darkness. At 65 d of age, quails were divided into four groups. One group of 25 quails was exposed to 22 ${}^{\circ }$C (thermoneutral group) and designated as the positive control. Four groups (100 quails) were exposed to 34 ${}^{\circ }$C for 8 h d${}^{-1}$ according to Mujahid et al. (2006) as heat-stressed groups and administered four dietary treatments including basal diet supplemented with DL-methionine to provide Met concentrations of 1.00 1.15, 1.30 and 1.45 times the quail's requirements per NRC (1994) recommendations (Met in the basal diet was calculated as the ingredients' composition which was reported by NRC). The thermoneutral and heat-stressed groups were divided into separate rooms. The birds were housed in galvanized wire cages equipped with feeders and nipple drinkers. Each cage provided a space of 0.2 m${}^{2}$ per five birds.

**Table 1:** Ingredients and chemical composition of the basal diet fed to breeder quails.

Ingredient	g/100 g
Corn	55.27
Soybean meal	32.50
Gluten meal	1.61
Vegetable oil	2.99
Carbonate calcium	5.64
Dicalcium phosphate	1.14
Common salt	0.34
Mineral premix${}^{1}$	0.25
Vitamin premix${}^{2}$	0.25
Calculated analysis	
ME${}^{3}$ (kcal kg${}^{-1}$)	2900
Crude protein (%)	20
Calcium (%)	2.5
Available phosphorus (%)	0.35
Sodium (%)	0.15
Arginine (%)	1.26
Lysine (%)	1.03
Methionine + cysteine (%)	0.70

${}^{1}$ Mixture supplied per kilogram of diet: retinyl acetate, 1.8 mg; cholecalciferol, 0.025 mg; DL-tocopheryl acetate, 1.25 mg; menadione sodium bisulfite, 2.5 mg; thiamine-hydrochloride, 1.5 mg; riboflavin, 3 mg; D-pantothenic acid, 5 mg; pyridoxine hydrochloride, 2.5 mg; vitamin B-12, 0.0075 mg; folic acid, 0.25 mg; niacin, 12.5 mg. ${}^{2}$ Mixture supplied per kilogram of diet: Mn (${\mathrm{MnSO}}_{4}H_{2}O$), 50 mg; Fe (${\mathrm{FeSO}}_{4}7H_{2}O$), 30 mg; Zn (ZnO), 30 mg; Cu (${\mathrm{CuSO}}_{4}5H_{2}O$), 5 mg; I (KI), 0.5 mg; Se (${\mathrm{Na}}_{2}{\mathrm{SeO}}_{3}$), 0.15 mg; Co (${\mathrm{CoCl}}_{2}6H_{2}O$), 0.1 mg; choline chloride, 125 mg. ${}^{3}$ ME: metabolizable energy.

Diets were formulated as shown in Table 1. The control diet was formulated mainly based on corn, soybean meal and corn gluten meal, and satisfied all NRC (1994) requirements. The birds had unlimited access to water and feed throughout the entire experiment period.

### 2.2 Performance trial of breeder quails

Egg production, feed intake, average egg weight (total weight of eggs laid/number of eggs laid, per experimental unit), feed conversion ratio (grams of feed per grams of eggs) and eggshell, yolk and albumen percentage were evaluated. Egg production was determined by daily egg collection, which was recorded on one spreadsheet per replicate. At 2, 4, 6 and 8 weeks of assay, all eggs produced in a single day were collected for quality evaluation (average weight, albumen and yolk percentages, eggshell thickness and Haugh unit). Average albumen weight per replicate was determined to be the difference between average egg weight and average yolk weight plus eggshell weight per replicate. In order to determine eggshell weight, eggs were cracked, and the shells were washed and dried at room temperature for 48 h prior to being weighed on an analytical balance. Shell thickness was measured by a micrometer as an average of three points (top, medial and base). The Haugh unit was calculated according to Brant et al. (1951), (100 log (H+7.57-1.7 W${}^{0.37}$)), where H is albumen height in millimeters and W is egg weight in grams.

Approximately 250 eggs (50 eggs per treatment) were collected over a 3 d period in week 8 and stored at 15 ${}^{\circ }$C before incubation. All eggs were incubated together. The setter was operated at a temperature of 37.7 ${}^{\circ }$C and a relative humidity of 60 % during the first 14 d of incubation, and eggs were turned once per hour. On day 15 of incubation, the eggs in each replicated tray (experimental eggs) were transferred to separate hatch baskets and placed in a hatcher for the final 3 d. The temperature was lowered to 37.2 ${}^{\circ }$C, and the relative humidity was raised to 70 %.

Unhatched eggs were analyzed for embryo mortality causes, which were classified as early, middle and late death. In order to determine offspring serum IgG (five blood samples per treatment), all 1 d old chicks were hatched from each replicate, bled by cardiac puncture, and blood was collected. The serum was then separated by centrifugation at 3000×g for 15 min (serum from each replicate was pooled). Samples were stored at -20 ${}^{\circ }$C.

### 2.3 Immune assay

#### 2.3.1 Egg yolk IgY purification

IgY was isolated from egg yolks using the Polson et al. (1980) method. Briefly, an equal volume of buffer (0.01 M sodium phosphate, 0.1 M NaCl, pH 7.5) was added to yolk and stirred. Solid polyethylene glycol 6000 (Sigma-Aldrich) was added to a concentration of 3.5 %, stirred until dissolved, and the resultant protein precipitate was pelleted by centrifugation at 10000×g for 15 min. The supernatant was filtered through filter paper and decanted into another centrifuge tube for further purification of IgY. Next, 12 % w/v solid polyethylene glycol (PEG) was added to the supernatant, stirred thoroughly and centrifuged at 10000×g for 15 min, in order to precipitate IgY. The pellet was redissolved in the original yolk volume in 0.01 M sodium phosphate buffer, 0.1 M NaCI, pH 7.5, and then PEG was added to 12 % w/v for a second precipitation. The supernatant was then decanted and the pellet was centrifuged twice more to remove any residual PEG trapped in the precipitate. This final IgY pellet was then dissolved in phosphate buffer (0.01 M, pH 7.5) to the original volume of yolk and stored at -20 ${}^{\circ }$C.

The total IgG levels in the maternal serum, IgY in the egg yolks and day-old chick's serum IgG were determined using sodium dodecyl sulfate polyacrylamide gel electrophoresis (SDS-PAGE) under reducing conditions according to Laemmli (1970). The resultant from the IgY precipitation steps was dissolved in the sample buffer with 2 % 2-mercaptoethanol and run on a 5 % stacking gel and 10 % separating gel. Samples and standards were loaded on the gel at a concentration of 20 µg. The gel was run at 20 mA for 1.5 h and stained with Coomassie Brilliant Blue. Densitometric analysis of the IgG and IgY was performed using RFLPscan software (Strategene, California, USA).

#### 2.3.2 Cutaneous basophil hypersensitivity

The cellular immune response was assessed by a cutaneous basophilic hypersensitivity test using phytohaemagglutinin (PHA). At day 30 and day 45 of the experiment, one quail from each replicate was selected, and the toe thickness of both the left and right feet at the third and fourth interdigital spaces was measured by micrometer. Immediately following the measurements, 0.05 mL of phosphate-buffered saline (PBS) and 0.05 mL PHA (1 mg PHA mL${}^{-1}$ in PBS) were injected intradermally into the foot webbing of the third and fourth digits of the right and left feet of each bird (left to act as the control), respectively. The web swelling of both feet were measured 12, 24 and 36 h after injection. The response was determined by subtracting the skin thickness of the first measurement from the second measurement, and values of the left foot (control) were subtracted from those of the right foot (Corrier and Deloach, 1990).

#### 2.3.3 Antibody titer to sheep red blood cells

Humoral immune response was evaluated by hemagglutination (HA) antibody titer estimation. A suspension of sheep red blood cell (SRBC) (1 % and 2.5 % v/v) in PBS was prepared and stored under refrigeration at 4 ${}^{\circ }$C until use. At days 30 and 45 of the experiment, 0.2 mL of 2.5 % SRBC suspension was injected intramuscularly per quail, and a total of five birds were injected per treatment in order to study the primary antibody response to SRBC. At days 37 and 52, respectively, for each SRBC injection, 1 mL of blood was collected from the wing vein. The blood was allowed to clot, and the serum was collected and frozen (-20 ${}^{\circ }$C) until analyzed for antibody titers to SRBC. The antibody titer was determined by HA test (Siegal and Gross, 1980) and titers were expressed as log${}_{2}$.

#### 2.3.4 White blood cell

Blood samples were collected from the wing vein of each bird into a bottle containing ethylene diamine tetra acetic acid (EDTA, 2 mg mL${}^{-1}$ of blood). Blood smears were fixed in alcohol and stained with Giemsa stain for differential white blood cell (WBC) counts. A total of 100 white cells were counted from each blood smear, and the percentage of each white cell type was calculated and multiplied by the total WBC count to obtain the absolute count. Heterophil/lymphocyte (H/L) ratios were calculated by dividing the number of heterophils by the number of lymphocytes (Gross and Siegel, 1983).

### 2.4 Assay of antioxidant indices and plasma lipids

At day 60, one quail from each replicate was randomly selected, and blood samples were taken and transferred to the animal house slaughter facility. Liver samples were taken immediately after slaughter and homogenized in freezing isotonic physiological saline to form homogenates at a concentration of 0.1 g mL${}^{-1}$. Liver samples were obtained to measure malondialdehyde (MDA) and antioxidant enzymes' activity.

Blood samples were collected from the wing vein into EDTA-coated syringes, and the plasma was separated by centrifugation at 3000×g for 15 min at room temperature and stored in aliquots at -20 ${}^{\circ }$C until use. The plasma samples were measured for MDA levels and for superoxide dismutase (SOD), catalase (CAT), glutathione peroxidase (GPx) and total antioxidant capacity (TAC).

Malondialdehyde levels and SOD, CAT and GPx were measured using spectrophotometric methods (Hitachi U-2001 spectrophotometer, Tokyo, Japan) as described in the literature. SOD activity was measured by the xanthine oxidase method, which monitors the inhibition of nitro blue tetrazolium reduction and the change of absorbance at 560 nm (Sun et al., 1988). CAT activity was measured following the decrease in absorbance at 240 nm due to hydrogen peroxide decomposition (Aebi, 1984). GPx activity was measured at 412 nm by quantifying the rate of oxidation of reduced glutathione to oxidized glutathione (Hafeman et al., 1974). The thiobarbital method (Placer et al., 1966) was used to determine the MDA level with a wavelength of 532 nm to determine absorbance.

Plasma levels of triglycerides, total cholesterol, low-density lipoprotein (LDL)-cholesterol and high-density lipoprotein (HDL)-cholesterol were analyzed using a spectrophotometer (Hitachi U-2001, Tokyo, Japan) per the instructions on the corresponding reagent kit (Pars Azmun, Tehran, Iran).

### 2.5 Statistical analysis

The obtained data were submitted to analysis of variance using the general linear model (GLM) procedure of the SAS statistical package (SAS Institute, 2002). Significant difference among means of treatments was detected by Duncan's multiple range test procedures. The differences were considered significant at P<0.05.

**Table 2:** Effect of methionine on egg weight, egg production, daily feed intake and FCR${}^{1}$ in breeder quails.

Parameter	Thermoneutral	Heat stress				SEM${}^{3}$	P value
		Basal diet	1.15 Met${}^{2}$	1.3 Met	1.45 Met		
Egg weight (g)	11.83${}^{c}$	11.58${}^{d}$	12.01${}^{b}$	12.39${}^{a}$	12.50${}^{b}$	0.022	<0.0001
Egg production (%)	76.94${}^{c}$	71.80${}^{d}$	81.00${}^{b}$	84.50${}^{a}$	85.00${}^{a}$	0.503	<0.0001
Daily feed intake (g)	24.84${}^{b}$	23.51${}^{c}$	25.23${}^{b}$	25.85${}^{a}$	26.16${}^{a}$	0.107	<0.0001
FCR	3.570${}^{a}$	3.690${}^{a}$	3.400${}^{b}$	3.240${}^{c}$	3.240${}^{c}$	0.041	<0.0001

${}^{1}$ FCR: feed conversion ratio. ${}^{2}$ Methionine. ${}^{3}$ SEM: standard error of means. ${}^{\text{a–c}}$ Means within rows with different superscripts are significantly different (P<0.05).

## 3 Results

### 3.1 Productive performance

The effects of HS on breeder quails were observed as a decrease in egg weight (EW), egg production (EP) and daily feed intake (DFI) in the HS group as compared to the thermoneutral (TN) conditions group (Table 2). Met supplementation increased EW, EP and DFI compared to the basal group exposed to HS. The feed conversion ratio (FCR) was significantly (P<0.05) improved by dietary supplementation with Met as compared to the basal group exposed to HS. Supplementation of the quail's diet with extra Met improved (P<0.05) EP, EW and DFI similar to those of the TN group.

**Table 3:** Effect of methionine on yolk and albumen percentage, shell thickness and Haugh unit in breeder quails.

Parameter	Thermoneutral	Heat stress				SEM${}^{2}$	P value
		Basal diet	1.15 Met${}^{1}$	1.3 Met	1.45 Met		
Yolk (%)	31.60${}^{a}$	30.42${}^{b}$	31.55${}^{a}$	32.02${}^{a}$	32.16${}^{a}$	0.140	0.001
Albumen (%)	57.27	58.51	58.42	58.51	58.19	0.138	0.064
Eggshell thickness (mm)	0.169${}^{b}$	0.164${}^{c}$	0.170${}^{b}$	0.175${}^{\mathrm{ab}}$	0.177${}^{a}$	0.002	0.001
Haugh unit	85.74${}^{a}$	81.32${}^{c}$	84.76${}^{b}$	85.10${}^{\mathrm{ab}}$	85.40${}^{\mathrm{ab}}$	0.194	<0.0001

${}^{1}$ Methionine. ${}^{2}$ SEM: standard error of means. ${}^{\text{a–c}}$ Means within rows with different superscripts are significantly different (P<0.05).

### 3.2 Egg quality parameters

The effects of dietary supplementation with Met during HS on egg quality parameters in Japanese breeder quails are presented in Table 3. There were no significant (P<0.05) differences in the yolk and albumen percentages across all experimental groups. The results showed that eggshell thickness and HU, an index of albumen quality, significantly (P<0.05) decreased in the HS group fed a basal diet as compared to the TN group. The results indicated that eggshell thickness and the HU score in groups that were fed experimental diets significantly (P<0.05) increased compared to the HS group. The eggshell thickness and HU score in Met supplementation groups were similar to those of the birds in the TN group.

**Table 4:** Effect of methionine on fertility, hatchability and embryonic mortality in breeder quails.

Parameter	Thermoneutral	Heat stress				SEM${}^{2}$	P value
		Basal diet	1.15 Met${}^{1}$	1.3 Met	1.45 Met		
Fertility (%)	89.69${}^{a}$	79.40${}^{b}$	87.65${}^{a}$	91.48${}^{a}$	88.86${}^{a}$	0.948	0.001
Hatchability (%)	76.78${}^{a}$	63.69${}^{b}$	76.71${}^{a}$	73.85${}^{a}$	72.22${}^{a}$	1.181	0.006
Embryonic mortality (%)							
Early death	3.062	7.144	6.532	5.307	5.716	0.644	0.381
Middle death	9.185	10.71	6.940	10.20	9.798	0.778	0.569
Late death	12.75	17.85	9.797	11.43	12.65	0.046	0.844
Total death	25.00${}^{b}$	35.71${}^{a}$	23.26${}^{b}$	26.93${}^{b}$	28.16${}^{b}$	1.154	0.011

${}^{1}$ Methionine. ${}^{2}$ SEM: standard error of means. ${}^{\text{a–c}}$ Means within rows with different superscripts are significantly different (P<0.05).

### 3.3 Fertility, hatchability and embryonic mortality

As shown in Table 4, fertility and hatchability decreased (P<0.05) in HS quails compared to those under TN conditions. Quails which were exposed to HS and fed with Met diets showed higher (P<0.05) fertility and hatchability than those fed the basal diet and exposed to the same HS conditions. Met inclusion in the diet of heat-stressed quails resulted in similar (P<0.05) fertility and hatchability compared to birds under TN conditions.

There was no significant (P<0.05) difference in early and middle embryonic mortality across all experiment groups. However, quails exposed to HS had higher (P<0.05) late and total embryonic mortality compared to quails in the TN group. All quails receiving Met in the diet had lower (P<0.05) late and total embryonic mortality compared to quails that were fed the basal diet and exposed to the same HS conditions. Supplementation of the quail's diet with Met resulted in similar (P<0.05) late and overall embryonic mortality compared to those reared under TN conditions.

**Table 5:** Effect of methionine on maternal serum IgG, egg yolk IgY, offspring serum IgG (mg mL${}^{-1}$) and IgG transfer${}^{1}$ in breeder quails.

Parameter	Thermoneutral	Heat stress				SEM${}^{3}$	P value
		Basal diet	1.15 Met${}^{2}$	1.3 Met	1.45 Met		
Maternal plasma IgG (mg mL${}^{-1}$)	11.80${}^{\mathrm{bc}}$	10.43${}^{c}$	13.13${}^{\mathrm{ab}}$	14.06${}^{a}$	14.33${}^{a}$	0.42	0.005
Egg yolk IgY (mg mL${}^{-1}$)	5.76${}^{\mathrm{bc}}$	4.36${}^{c}$	6.37${}^{a}$	6.54${}^{a}$	6.77${}^{a}$	0.30	0.040
Offspring serum IgG (mg mL${}^{-1}$)	3.80${}^{\mathrm{bc}}$	2.43${}^{c}$	4.60${}^{b}$	5.11${}^{\mathrm{ab}}$	5.90${}^{a}$	0.36	0.003
IgG transfer (%)	32.20	23.29	35.03	36.34	41.17	–	–

${}^{1}$ The percentage transfer of IgG from the dams' plasma to the plasma of 1 d old chicks was calculated by dividing the 1 d old chicks'plasma antibody levels by the dams' plasma antibody levels and multiplying this value by 100. ${}^{2}$ Methionine. ${}^{3}$ SEM: standard error of means. ${}^{\text{a–c}}$ Means within rows with different superscripts are significantly different (P<0.05).

### 3.4 Immune assay

#### 3.4.1 Immunoglobulins and IgY transfer to offspring

Results cited in Table 5 show that the maternal serum IgG, egg yolk IgY, offspring serum IgG and IgG transfer to offspring in breeder quails fed diets supplemented with Met under hot environmental stress. Results indicated that maternal serum IgG, egg yolk IgY, offspring serum IgG and IgG transfer to offspring significantly (P<0.05) decreased in the quails fed the basal diet and exposed to HS as compared to the TN group. The results indicated that maternal serum IgG, egg yolk IgY and offspring serum IgG in groups fed the experimental diets (HS plus Met supplementation) increased significantly (P<0.05) compared to the HS group. The maternal serum IgG, egg yolk IgY and offspring serum IgG in HS plus Met concentrations of 1.15 and 1.30 times the quail's NRC (1994) recommendations groups were similar to those of the birds in the TN group. In addition, these three factors in HS plus the 1.45 times Met groups were significantly (P<0.05) higher than those same factors in the TN group.

**Table 6:** Effect of methionine on humoral (log${}_{2}$) and cell immune response in breeder quails.

Parameter	Thermoneutral	Heat stress				SEM${}^{4}$	P value
		Basal diet	1.15 Met${}^{1}$	1.3 Met	1.45 Met		
SRBC${}^{2}$							
Primary injection	2.33${}^{a}$	1.33${}^{b}$	2.40${}^{a}$	2.60${}^{a}$	2.60${}^{a}$	0.28	0.04
Secondary injection	5.25${}^{a}$	3.25${}^{b}$	5.20${}^{a}$	5.40${}^{a}$	5.80${}^{a}$	0.28	0.02
PHA${}^{3}$ (mm)							
Primary PHA response							
12 h	0.184${}^{a}$	0.113${}^{b}$	0.187${}^{a}$	0.206${}^{a}$	0.205${}^{a}$	0.007	<0.0001
24 h	0.176${}^{a}$	0.106${}^{b}$	0.177${}^{a}$	0.196${}^{a}$	0.214${}^{a}$	0.010	0.0030
36 h	0.170${}^{a}$	0.099${}^{b}$	0.184${}^{a}$	0.186${}^{a}$	0.194${}^{a}$	0.006	<0.0001
Secondary PHA response							
12 h	0.231${}^{a}$	0.111${}^{b}$	0.216${}^{a}$	0.224${}^{a}$	0.221${}^{a}$	0.014	0.012
24 h	0.227${}^{a}$	0.107${}^{b}$	0.189${}^{a}$	0.183${}^{a}$	0.217${}^{a}$	0.015	0.011
36 h	0.208${}^{a}$	0.098${}^{b}$	0.182${}^{a}$	0.180${}^{a}$	0.210${}^{a}$	0.014	0.006

${}^{1}$ Methionine. ${}^{2}$ Sheep red blood cell. ${}^{3}$ Phytohaemagglutinin. ${}^{4}$ SEM: standard error of means. ${}^{\text{a–b}}$ Means within rows with different superscripts are significantly different (P<0.05).

#### 3.4.2 Humoral and cell mediate immune

The data in Table 6 represent the humoral immune responses and cell-mediated immune responses of birds. Birds reared under HS conditions showed a significant (P<0.05) reduction in antibody titers against SRBC during primary and secondary responses. The results indicated that dietary Met significantly increased titers of antibodies for both primary and secondary responses when birds were exposed to HS conditions (P<0.05), whereas antibody titers against SRBC were not affected by the Met supplementation as compared to the TN treatment (P<0.05).

The cell-mediated immune response to PHA was reduced (P<0.05) by HS. Supplementation of Met increased (P<0.05) stimulation indices 12 and 24 h after the injection of PHA compared to the basal group which was exposed to HS and resulted in a similar response to PHA compared to quails under TN conditions.

**Table 7:** Effect of methionine on heterophil, lymphocyte, monocyte, eosinophil and H/L${}^{1}$ ratios in breeder quails.

Parameter	Thermoneutral	Heat stress				SEM${}^{3}$	P value
		Basal diet	1.15 Met${}^{2}$	1.3 Met	1.45 Met		
Heterophil (%)	26.6${}^{c}$	34.66${}^{a}$	31.20${}^{\mathrm{bc}}$	29.0${}^{\mathrm{bc}}$	26${}^{c}$	0.78	0.005
Lymphocyte (%)	71.6${}^{a}$	64.0${}^{c}$	67.2${}^{\mathrm{bc}}$	68.8${}^{\mathrm{ab}}$	71.6${}^{a}$	0.73	0.001
Monocyte (%)	0.30	1.00	0.60	1.20	1.0	0.10	0.6
Eosinophil (%)	1.30	0.60	1.00	1.00	1	0.20	0.8
H/L	0.37${}^{c}$	0.54${}^{a}$	0.46${}^{b}$	0.42${}^{\mathrm{bc}}$	0.36${}^{c}$	0.01	0.003

${}^{1}$ Heterophil/lymphocyte.${}^{2}$Methionine. ${}^{3}$SEM: standard error of means. ${}^{\text{a–c}}$ Means within rows with different superscripts are significantly different (P<0.05).

#### 3.4.3 White blood cells

The effects of dietary treatments on WBC are shown in Table 7. HS led to an increase in heterophil and H/L ratio and a decrease in lymphocyte and eosinophil as compared to the TN group (P<0.05). All breeder quails receiving Met treatments had a lower (P<0.05) heterophil and H/L ratio as well as higher (P<0.05) lymphocytes than quails fed the basal diet under the same stress conditions. The heterophil, lymphocyte and H/L ratio in HS plus the 1.30 and 1.45 times Met groups were similar to those of the birds in the TN group. Monocyte and eosinophil did not differ among treatments throughout the study (P<0.05).

**Table 8:** Effect of methionine on antioxidant parameters and malondialdehyde in the plasma and liver of breeder quails.

Parameter	Thermoneutral	Heat stress				SEM${}^{7}$	P value
		Basal diet	1.15 Met${}^{1}$	1.30 Met	1.45 Met		
Plasma							
SOD${}^{2}$ (unit mL${}^{-1}$)	164${}^{d}$	154${}^{e}$	170${}^{c}$	179${}^{b}$	207${}^{a}$	4.12	<0.0001
CAT${}^{3}$ (unit mL${}^{-1}$)	6.64${}^{c}$	4.76${}^{d}$	6.52${}^{c}$	6.84${}^{b}$	7.58${}^{a}$	0.19	<0.0001
GPx${}^{4}$ (unit mL${}^{-1}$)	185${}^{d}$	173${}^{e}$	196${}^{c}$	205${}^{b}$	255${}^{a}$	3.87	<0.0001
MDA${}^{5}$ (nmol m${}^{-1}$)	3.48${}^{c}$	3.71${}^{a}$	3.58${}^{b}$	3.49${}^{c}$	3.40${}^{d}$	0.02	<0.0001
TAC${}^{6}$ (mmol L${}^{-1}$)	5.90${}^{c}$	4.12${}^{d}$	5.08${}^{d}$	6.19${}^{b}$	6.90${}^{a}$	0.20	<0.0001
Liver							
SOD (unit mL${}^{-1}$)	120${}^{c}$	113${}^{d}$	120${}^{c}$	132${}^{b}$	148${}^{a}$	2.81	<0.0001
CAT (unit mL${}^{-1}$)	22.0${}^{b}$	16.9${}^{c}$	22.8${}^{b}$	23.4${}^{b}$	24.5${}^{a}$	0.55	<0.0001
GPx (unit mL${}^{-1}$)	21.5${}^{c}$	19.5${}^{d}$	21.6${}^{c}$	23.2${}^{b}$	25.4${}^{a}$	0.44	<0.0001
MDA (nmol m${}^{-1}$)	0.97${}^{c}$	1.46${}^{a}$	1.41${}^{\mathrm{ab}}$	1.45${}^{a}$	1.38${}^{b}$	0.03	<0.0001

${}^{1}$ Methionine. ${}^{2}$ Superoxide dismutase. ${}^{3}$ Catalase. ${}^{4}$ Glutathione peroxidase. ${}^{5}$ Malondialdehyde. ${}^{6}$ Total antioxidant capacity. ${}^{7}$ SEM: standard error of means. ${}^{\text{a–c}}$ Means within rows with different superscripts are significantly different (P<0.05).

### 3.5 Antioxidant enzyme activities and lipid peroxidation

Table 8 shows the effects of dietary treatments on plasma and liver antioxidant indices. The plasma and liver activities of SOD, CAT and GPx were lower (P<0.05) in the HS group compared to the TN group, whereas the reverse was true for malondialdehyde concentrations. Dietary supplementation with Met increased (P<0.05) the plasma and liver activity of SOD, CAT and GPx, and reduced (P<0.05) the concentrations of MDA. In addition, the administration of 1.15 times the NRC (1994) recommended Met requirements to the diet increased (P<0.05) the plasma and liver activities of SOD, CAT and GPx and reduced (P<0.05) the concentrations of MDA in heat-stressed quails to levels similar to those for quails under TN conditions. Quails receiving diets of 1.30 and 1.45 times the Met had higher (P<0.05) plasma and liver activity of SOD, CAT and GPx, and lower levels of MDA compared to the TN group. Breeder quails receiving the Met diet exhibited a higher (P<0.05) plasma TAC compared to the HS and TN groups.

**Table 9:** Effect of methionine on plasma lipid parameters in breeder quails.

Parameter	Thermoneutral	Heat stress				SEM${}^{2}$	P value
		Basal diet	1.15 Met${}^{1}$	1.3 Met	1.45 Met		
HDL-cholesterol (mmol L${}^{-1}$)	3.72${}^{a}$	3.17${}^{b}$	3.57${}^{a}$	3.60${}^{a}$	3.70${}^{a}$	0.063	0.01
LDL-cholesterol (mmol L${}^{-1}$)	2.33${}^{b}$	2.99${}^{a}$	2.47${}^{b}$	2.34${}^{b}$	2.23${}^{b}$	0.078	0.034
Total cholesterol (mmol L${}^{-1}$)	4.18${}^{b}$	5.00${}^{a}$	3.84${}^{b}$	3.90${}^{b}$	4.06${}^{b}$	0.113	0.009
Triglycerides (mmol L${}^{-1}$)	2.15${}^{b}$	2.47${}^{a}$	2.13${}^{b}$	2.22${}^{b}$	1.99${}^{b}$	0.042	0.01

${}^{1}$ Methionine. ${}^{2}$ SEM: standard error of means. ${}^{\text{a–b}}$ Means within rows with different superscripts are significantly different (P<0.05).

### 3.6 Plasma lipids

Table 9 shows the effects of dietary treatments on plasma levels of HDL-cholesterol, LDL-cholesterol, total cholesterol and triglycerides. Results indicated that the HDL-cholesterol was lower (P<0.05) and LDL-cholesterol, total cholesterol and triglycerides were higher (P<0.05) in the HS group compared to the TN group. All quails receiving Met in the diet had lower (P<0.05) plasma levels of triglycerides, total cholesterol and LDL-cholesterol as well as higher (P<0.05) plasma levels of HDL-cholesterol compared to the quails fed the basal diet and exposed to the same HS condition. Supplementation of the quail's diet with Met resulted in plasma lipid levels (P<0.05) similar to those reared under TN conditions.

## 4 Discussion

In the current study, high environmental temperature reduced the DFI, EP, EW and egg quality, which is in agreement with previous findings (Tables 2, 3). The reduced EP and EW could be attributed in part to the decrease in feed intake and to an impairment in the utilization of nutrients. Heat stress could also negatively impact nutrient utilization in poultry via changes in the intestinal morphology, where different intestinal segments (duodenum, jejunum and ileum) exhibit lesions that vary in their degree, as well as differences in their relative weight, villus height, villus surface area, crypt depth (Al-Fataftah and Abdelqader, 2014), immunoglobulin A-secreting cell area and epithelial cell area (Lambert et al., 2002) and by decreasing the activity of digestive enzymes (Ruan and Niu, 2001; Yi et al., 2016).

When the ambient temperature exceeds the thermoneutral zone for birds, feed intake is decreased in order to minimize the production of metabolic heat (Swennen et al., 2007), which adversely affects productive performance (Quinteiro-Filho et al., 2010).

To minimize these adverse effects of HS, many practical approaches have been suggested to enhance the thermotolerance of birds in order to support productivity (Del Vesco et al., 2014). In our study, we investigated Met as a way to protect quails against HS. Diet supplementation with 1.15, 1.30 and 1.45 times the Met requirements restored the impairment in DFI, EP, EW and egg quality in quails submitted to HS. The reduced EP and egg quality in heat-exposed quails might be due to the reduction in feed intake and an impairment in the utilization of nutrients. In addition, diet supplementation with Met improves productive performance through pathways of polyamine metabolism (Gonzalez-Esquerra and Leeson, 2006). Glutathionine derived from Met may also reduce damage from oxidative stress. Therefore, the TSAA requirement would be higher under hyper-thermoneutral conditions than under thermoneutral conditions. The inclusion of a high level of Met (1.30 and 1.45 times the Met requirement) improved productive performance in the HS group compared to the TN group. Dietary supplementation with Met for breeder quails exposed to HS significantly improved the egg parameters including egg weight and HU scores to levels similar to those of quails reared under TN conditions. Several investigators also reported significant increases in egg production with higher Met + Cys levels (Dabbert et al., 1996). It was suggested that increased Met + Cys levels improved egg weight and, subsequently, feed efficiency. Birds exposed to HS conditions showed decreased feed intake, decreased feed efficiency and a consequent drop in egg production and deteriorated egg quality (Austic, 1985; Bunchasak and Silapasorn, 2005). Star et al. (2009) reported a reduction of 31.6 %, 36.4 % and 3.41 % in the feed conversion ratio, egg production and egg weight, respectively, in laying hens exposed to HS conditions. It is obvious that dietary Met supplementation could counter the adverse effects of HS with regard to egg yield.

The internal quality of eggs can be evaluated particularly by HU. Birds exposed to HS and fed the basal diet had the lowest HU score compared to other HS groups. Thus, it can be deduced that Met concentrations of 1.15, 1.30 and 1.45 times the NRC (1994) recommendations in breeder quail diets under a heat-stressed environment were sufficient to improve the quality of albumen as evidenced by HU scores to a level similar to that of quails reared under TN conditions.

Heat stress can affect the reproductive function of poultry in different ways. Heat-stressed birds had depressed egg production due to imponderables in the calcium–estrogen relationship, so we can infer that HS decreases albumin consistency, yolk size and calcium deposits in the egg shell. The depression in the ovarian blood flow is a possible mechanism for the reduction in ovarian function as a differential ovarian blood flow pattern was detected in laying hens subjected to HS (Mashaly et al., 2004). Dietary supplementation with Met in breeder quails exposed to HS significantly improved the egg parameters including egg weight, eggshell thickness, albumen percentage and yolk percentage. Consequently, Met could eliminate the negative effects of HS with regard to hatchability.

The results of this study showed that the embryonic mortality of breeder quails decreased by dietary supplementation with Met in the HS group. Tolba et al. (2015) reported that Met supplementation in the diet of quails can improve the infertility egg ratio, early embryonic mortality and later embryonic mortality. In this context, the reduction in mortality in Met group quails may also be attributed to improved immunity as discussed below.

In addition to impairing performance, HS also affects the immune response in birds. In the current study, Met improved the maternal serum IgG, egg yolk IgY and offspring serum IgG in comparison to the basal group exposed to thermal stress. The levels of supplementation can also strongly affect immunomodulation. In the present study, higher levels of Met fed to heat-stressed quails resulted in higher levels of maternal serum IgG, egg yolk IgY and offspring serum IgG compared to similar parameters in TN quails. The highest antibody transmission rate from breeder quails to offspring also belonged to quails that received 1.45 times the Met requirement. The percentage of IgY transfer can be expressed as the percentage of the dam's plasma IgY levels circulating in the blood of 1 d old chicks (approximately 33.60 %). In agreement with our results, Loeken and Roth (1983) reported that the amount of IgY transported is independent of egg size and known to be proportional to the maternal serum IgY concentration.

In this study, the humoral and cell-mediated immune responses were suppressed under HS conditions. It is well known that environmentally stressed poultry generally have a depressed humoral immune response (Gharib et al., 2005). In agreement with the present study, Met supplementation optimized the response to PHA as well as total antibody response to SRBC as a T-dependent antigen (Tsiagbe et al., 1987). The Met level to optimize leukocyte migration inhibition assay was also higher than the level to improve growth in broiler chicks (Kidd, 2004). Inclusion of Met in the diet restored either humoral or cell-mediated immune responses to levels similar to those for breeder quails submitted to TN. The improved immune response in breeder quails fed with Met could be attributed to Met's role as a precursor to Cys. There are some reports that supplementation of poultry diets with higher levels of SAA improves immune responses (Swain and Johri, 2000).

The heterophil-to-lymphocyte ratio has been used as a sensitive indicator of stress, including HS, in poultry (Gross and Siegel, 1983; Mashaly et al., 2004). An increased H/L ratio was observed under high ambient temperature, which demonstrates an association between HS and non-specific immune reactive cells (Mashaly et al., 2004). It has been shown that HS causes an increase in the H/L ratio due to reduced numbers of circulating lymphocytes and higher numbers of heterophils (Prieto and Campo, 2010; Felver-Gant et al., 2012). The higher population of WBC in the peripheral blood by the dietary Met after stress was in agreement with the results of Al-Mayah (2006), who reported that manipulation of dietary nutrients resulted in immunoregulatory consequences due to the involvement of the nutrient or its products in communication within and between leukocytes. Payne et al. (1990) and Bhargava et al. (1971), respectively, showed that a deficiency or surplus of dietary protein or amino acids can alter immune responses. It has been shown that Met interacts with the immune system, improving both humoral and cellular response. Also, Met requirements must be higher when the goal is to maintain optimal immunity levels, as compared to growth (Swain and Johri, 2000; Shini et al., 2005) and that lower sulfur amino acid levels result in a severe lymphocyte depletion in intestinal tissue (Peyer's patches) and in the lamina propria (Swain and Johri, 2000).

Heat stress could also induce oxidative stress resulting in an imbalance in antioxidant status in birds (Lin et al., 2006). Under HS conditions, as the bird's body attempts to maintain its thermal homeostasis, increased levels of ROS occur. Consequently, tissues and cells possess defense mechanisms to detoxify ROS by radical scavengers such as SOD, CAT and GPx (Wu et al., 2004). The results of our study are in agreement with data available in the literature. In the present study, the activities of SOD, CAT and GPx were lower and MDA concentration in serum was higher in quails exposed to HS compared to the TN group. Dietary Met supplementation increased the serum and liver activity of antioxidant enzymes (SOD, CAT and GPx) and reduced the concentrations of MDA, an indirect parameter of lipid peroxidation and overproduction of ROS, in heat-stressed quails. Inclusion of the higher level (1.45 times) of Met in the diet resulted in higher levels of antioxidant enzymes and lower MDA than in the TN group. Synthesizing antioxidant enzymes such as SOD and GPx is an important regulation with regard to animal responses to stress conditions. Glutathione is a tripeptide synthetized from glutamate, glycine and cysteine. Cysteine can be synthetized from homocysteine from the precursor Met (Shoveller et al., 2005); therefore, when dietary Met is available in adequate amounts, larger quantities can be directed towards cysteine synthesis via the transsulfuration pathway.

With regard to HDL and LDL plasma levels, the present study showed a positive trend for an increase in HDL and a decrease in LDL in supplemented Met (1.15, 1.30 and 1.45 times the Met requirements as NRC (1994) recommended) vs. the HS group fed a basal diet. This indicates the beneficial and protective effects of these methyl donors on bird health in particular and on productive performance in general. In addition, administration of Met to the diet restored plasma levels of HDL-cholesterol, LDL-cholesterol, total cholesterol and triglycerides near to those of the TN group.

Possible mechanisms for the lipid-lowering effects of Met might be related to its antioxidant properties. In our study, Met supplementation reduced serum peroxide content. The maintenance of the normal structure of lipoprotein receptors is necessary for their function, improving the cellular uptake of serum lipids from the blood. Reactive oxygen species produced during oxidative stress react with lipoproteins to produce oxidation states, decreasing the cellular uptake of lipids from the blood (Diniz et al., 2006; Schaffer, 2003; Brizzi et al., 2003). Thus, the antioxidant action of Met might contribute to elevated cellular lipid uptake, resulting in a decrease in serum cholesterol levels.

## 5 Conclusion

Breeder quails subjected to the stress of a high thermal environment exhibited deteriorated productive performance, reproduction, egg quality, antioxidant status and immune response. Dietary supplementation with either 1.15, 1.30 and 1.45 times the Met requirements (as NRC recommendation) in breeder quails reared under HS conditions can alleviate the adverse effects of HS and improve the performance, reproduction, antioxidant status and immunity, as well as maternal antibody transmission. In addition, administration of Met to the diet restored most parameters nearer to the TN group. In some cases, supplementation of the diet with 1.45 times the Met requirement resulted in better responses compared to quails under TN conditions. Therefore, it was concluded that Met in higher levels of recommended NRC (1994) could be used as a feed additive in breeder quails for the alleviation of the adverse effects of HS.

## Data Availability

The original data of the paper are available upon request from the corresponding author.
